# Supplementary material for: Near-isotropic super-resolution microscopy with axial interference speckle illumination
Source: Nat Commun. 2025 Oct 20;16:9274. doi: 10.1038/s41467-025-64366-2 (PMC12537971; doi:10.1038/s41467-025-64366-2)
Supplement: Supplementary file 1 — Supplementary Information [file 41467_2025_64366_MOESM1_ESM.pdf]

Near-isotropic Super-Resolution Microscopy with Axial Interference Speckle Illumination

Supplementary Information

Hajun Yoo[1], Kwanhwi Ko[1], Sukhyeon Ka[1], Gwiyeong Moon[1], Hyunwoong Lee[1], Seongmin Im[1], Peng Xi[2] and Donghyun Kim[1,3]*

[1]School of Electrical and Electronic Engineering

Yonsei University, Seoul, Korea 03722

[2]Department of Biomedical Engineering

National Biomedical Imaging Center, College of Future Technology

Peking University, Beijing, China 100871

[3]Department of Biomedical Engineering

The Chinese University of Hong Kong, Shatin, N.T., Hong Kong

* Corresponding author: kimd@yonsei.ac.kr

Supplementary Notes

Supplementary Note 1 | Numerical Simulations

3D finite-difference time-domain (FDTD) simulations were conducted to calculate the speckle patterns generated by the interaction of incident light with and without a mirror. A series of randomly polarized plane-wave light sources, each with a wavelength of 488 nm, were modeled at random incident angles (up to a maximum of 79.5°) to simulate light entering through an objective lens with a Numerical Aperture (NA) of 1.49. Each plane wave was assigned to random amplitude and phase values to ensure that the superposition of these waves was computed within the FDTD simulation region. To accurately simulate the electromagnetic behavior, perfectly matched layer boundary conditions were applied along all three axes (x, y, and z directions) to prevent reflections and ensure that the electromagnetic waves propagated freely across the simulation boundaries. The refractive index (RI) value used in the simulation was 1.515 for both the immersion oil and glass substrates, with the values for the water layer sourced from Palik[1] (n = 1.340 at λ = 488 nm, 1.337 at λ = 532 nm, and 1.332 at λ = 633 nm) and the complex refractive indices for the silver mirror from Johnson and Christy[2] ($n + ki$ = 0.050 + 3.024i at λ = 488 nm, 0.054 + 3.434i at λ = 532 nm, and 0.056 + 4.285i at λ = 633 nm).

Supplementary Note 2 | Detailed theory of DSI and AXIS illumination

By introducing ρ as the fluorescence density of the sample, h^{3D} as the 3D point spread function (PSF) of the optical system, and $I_s^{3D}(t)$ as the speckle illumination at a given time t, the theoretical description of detection intensity of the camera F is described as

$$F(r_d, z_d, t) = \iiint h^{3D}(r_d - r, z_d - z)\rho(r, z)I_s^{3D}(r, z, t)dr^2dz \quad (1)$$

At the z_c focal plane, the camera reads the following signal

$$F(r_d, z_c, t) = \iint h^{3D}(r_d - r, 0)\rho(r, z_c)I_s^{3D}(r, z_c, t)dr^2 \quad (2)$$

As described in the Methods in main text, the Richardson-Lucy (RL) algorithm was used for deconvolution, enhancing both image resolution and contrast as demonstrated below.

$$F_{\mathrm{RL}}(r_d, z_c, t) = \iint h_{\mathrm{RL}}^{3D}(r_d - r, 0)\rho(r, z_c)I_s^{3D}(r, z_c, t)dr^2 \quad (3)$$

Through deconvolution, the PSF is replaced by h_{RL}^{3D}.

The variance of the images obtained in z_c focal plane is defined as follows[3]:

$$V^{2D}(r_d, z_c) = \langle F_{\mathrm{RL}}(r_d, z_c, t)^2\rangle_t - {\langle F_{\mathrm{RL}}(r_d, z_c, t)\rangle_t}^2 \quad (4)$$

Since $\iint h_{\mathrm{RL}}^{3D}(r, 0)dr^2 = A$, A is a constant, and $\langle I_s^{3D}(r, z, t)\rangle_t = \langle I_s^{3D}\rangle$,which is also constant, the first part of the Supplementary Equation (4) can be simplified to Supplementary Equation (5). This simplification also uses the autocorrelation relation $\langle I_s^{3D}(r, z_c, t) \cdot I_s^{3D}(r', z_c, t)\rangle_t = \langle I_s(r, z_c)\rangle^2\{1 + h_{ill}^{3D}(\Delta r, 0)\}$and $R_{\mathrm{RL}}^{3D}(\Delta r, 0) = \int h_{\mathrm{RL}}^{3D}(r_d - r, 0) \cdot h_{\mathrm{RL}}^{3D}(r_d - r + \Delta r, 0)dr^2$.

$$\begin{aligned}
&\langle F_{\mathrm{RL}}(r_d, z_c, t)^2\rangle_t \\
&= \iiint\iiint h_{\mathrm{RL}}^{3D}(r_d - r_i, 0) \cdot h_{\mathrm{RL}}^{3D}(r_d - r_k, 0) \cdot \rho(r_i, z_c) \cdot \rho(r_k, z_c) \cdot \langle I_s^{3D}(r, z_c)\rangle^2 \\
&\qquad \cdot \{1 + h_{ill}^{3D}(\Delta r, 0)\}dr_i^2 dr_k^2 \\
&= A^2\rho^2\langle I_s^{3D}\rangle^2 + \rho^2\langle I_s^{3D}\rangle^2 \int R_{\mathrm{RL}}^{3D}(\Delta r, 0)h_{ill}^{3D}(\Delta r, 0)dr^2 \quad (5)
\end{aligned}$$

The second part of the Supplementary Equation (4) is

$${\langle F_{\mathrm{RL}}(r_d, z_c, t)\rangle_t}^2 = A^2\rho^2\langle I_s^{3D}\rangle^2 \quad (6)$$

Thus, the final variance can be concisely expressed as shown in the following Supplementary Equation (7), where the root mean square (RMS) at the camera plane becomes proportional to the fluorescence density of the sample as Supplementary Equation (8).

$$V^{2D}(r_d, z_c) = \rho^2 \langle I_s^{3D} \rangle^2 \int R_{\mathrm{RL}}^{3D}(\Delta r, 0) h_{ill}^{3D}(\Delta r, 0) dr^2 \tag{7}$$

$$RMS(r_d, z_c) = \rho \langle I_s^{3D} \rangle \sqrt{\int R_{\mathrm{RL}}^{3D}(\Delta r, 0) h_{ill}^{3D}(\Delta r, 0) dr^2} \tag{8}$$

Two key findings can be drawn from the Supplementary Equation (8):

- The value of the $R_{\mathrm{RL}}^{3D}(\Delta r, 0)$, defined as the autocorrelation of detection PSF, shows that the volume of the detection PSF has decreased due to the RL deconvolution process. This indicates that deconvolution has improved the optical sectioning capability, which reduces out-of-focus signals and is realized within the dynamic speckle illumination (DSI) microscopy framework.
- Since $h_{ill}^{3D}(\Delta r, 0)$ represents the illumination PSF, defined as the speckle illumination PSF in the main text, it can be significantly reduced in the z-direction by axial interference speckle (AXIS) illumination. This further indicates that the optical sectioning capability has been enhanced more than four-fold in the z-direction, as supported by the numerical evidence in the main text, within the DSI framework.

Based on the two findings mentioned, DSI inherently achieves superior optical sectioning performance with AXIS illumination compared to conventional DSI with random speckle illuminations. Therefore, we introduced the intensity weighting function to the pre-RL deconvolved image set F_{RL}. This ensures that the image set F' contains higher in-focus fluorescence signals before proceeding with the cumulant calculation (described in the main text Methods).

Supplementary Note 3 | Axial distortion correction

Although our motorized nanostage allowed precise control of the z-axis step, axial distortion inevitably occurred due to the mismatch between the RI of the substrate, immersion oil, and sample medium[4]. This mismatch resulted in deviation between the actual z-stage movement and the imaging depth. We corrected for this distortion in entire experiments using the well-known axial distortion correction factor, as described by Supplementary Equation (9):

$$\frac{d'}{d} = \frac{\tan\left(\sin^{-1}\frac{0.5NA}{n_1}\right)}{\tan\left(\sin^{-1}\frac{0.5NA}{n_2}\right)} \tag{9}$$

Here, d', d represents the actual and expected focal position, and NA is the numerical aperture of the objective lens, with n_1 and n_2 being the refractive indices of the immersion medium and the sample medium, respectively. This formula is derived using the median ray method[4]. Based on the corrected axial distortion, the PSF used for the reconstruction is also adjusted accordingly as described in Supplementary Table 4. The experimental comparison of the axial distortion correction before and after adjustment is presented in Supplementary Fig. 2a, b.

Supplementary Note 4 | Mirror height and laser coherence length

When the mirror–sample separation increases, the interference contrast (i.e., visibility) inevitably decreases. The drop becomes significant only when the optical-path difference (OPD) exceeds the temporal coherence length of the illumination source. To quantify this effect, we calculated the visibility as a function of mirror height d for a 488 nm diode laser.

The OPD introduced by the mirror is

$$OPD = 2nd, \tag{10}$$

where n the refractive index of the medium. For a gaussian-spectrum source with coherence length L_c, the field correlation envelope is

$$\gamma(d) = \exp\left(-\frac{(OPD)^2}{2L_c^2}\right), \tag{11}$$

and the resulting visibility follows

$$V(d) = \frac{2\gamma(d)}{1+\gamma(d)^2} \ . \tag{12}$$

A detailed derivation of these relations is described in ref. [5].

Using typical coherence lengths reported for diode lasers (L_c, on the order of 1–10 mm), we evaluated $V(d)$ over a range of mirror heights (see Supplementary Fig. 11). The calculations show that, for diode sources, the visibility begins to decay markedly once mirror height exceeds ≈ 500 μm, in agreement with previous studies[6]. By contrast, gas lasers such as the HeNe—whose coherence lengths are an order of magnitude longer—maintain high visibility over millimeter-scale separations. In our experimental setup, the mirror is positioned less than 100 μm above the sample, separated by a thin water layer. This distance keeps the OPD well within the laser coherence length, ensuring that interference contrast is preserved.

Supplementary Note 5 | FRC-assisted AXIS-SIM and z-Intensity Flattening

In our original workflow, RL-deconvolution was applied with fixed iteration numbers—7 for pre-deconvolution and 8 for post-deconvolution—selected conservatively based on preliminary visual assessment and literature precedent to avoid over-deconvolution. However, to objectively determine the optimal number of iterations during RL-deconvolution, we then implemented a Fourier ring correlation (FRC)-assisted stopping criterion. FRC provides a resolution metric by comparing spatial-frequency content between two statistically independent images and has been employed in determining the iteration of deconvolution by estimating a reliable cutoff frequency[7]. As reported, excessive iterations in RL-deconvolution can lead to a high-frequency noise amplification (so-called noise blow-up) without delivering further resolution gains[8]. Accordingly, we kept the pre-deconvolution iteration fixed at an early stage (seven iterations) and applied our FRC-based stopping criterion only during post-deconvolution to select its optimal iteration number. For each iteration, the reconstructed z-stack was split into odd- and even-indexed frames, which were then merged to produce two independent subsets. Maximum intensity projections of these subsets were used to compute 2D FRC curves, and the spatial frequency at which the curve crossed the 1/7 threshold was recorded. While this approach provides an effective approximation for evaluating lateral resolution trends, it does not capture the full volumetric resolution. Because the analysis is based on 2D projections rather than the 3D distribution of signal and noise, the resulting FRC values should be interpreted as a projected-resolution estimate rather than a true 3D resolution measurement. As shown in Supplementary Fig. 15a, while the nominal FRC resolution improved with increasing iterations (e.g., 103.2 nm at 9 iterations, 87.2 nm at 20, and 79.6 nm at 30), we also observed the noise blow-up effect beyond a certain point. We therefore stopped at the iteration where the FRC-estimated resolution first saturated and before any noise amplification appeared. Applying this protocol, we found that the optimal number of post-deconvolution iterations was 9 for the microtubule data (Supplementary Fig. 15a) and 3 for the bead dataset (Supplementary Fig. 16). To further improve image uniformity along the z-axis, we applied a z-intensity flattening procedure. Due to illumination inhomogeneities or signal attenuation in thick specimens, the mean intensity often varies across z-planes, which can introduce stripe-like artifacts in projections or depth-resolved views. To correct this, we computed the mean z-intensity profile of the DL image stack and applied a low-pass filter to extract the global z-trend. A slice-wise correction factor was then calculated as the ratio of the original to the smoothed profile and used to normalize each z-slice (Supplementary Fig. 15b). This flattening procedure improved visual consistency across depths and mitigated z-dependent artifacts, as demonstrated in representative images (Supplementary Fig. 15c). However, this process primarily enhances the visual appearance and had minimal impact on actual resolution improvement (less than 1% as quantified in Supplementary Table 5). These results indicate that the procedure mainly reduces stripe artifacts and does not alter the validity of the main dataset presented in the primary figures.

Supplementary Figures

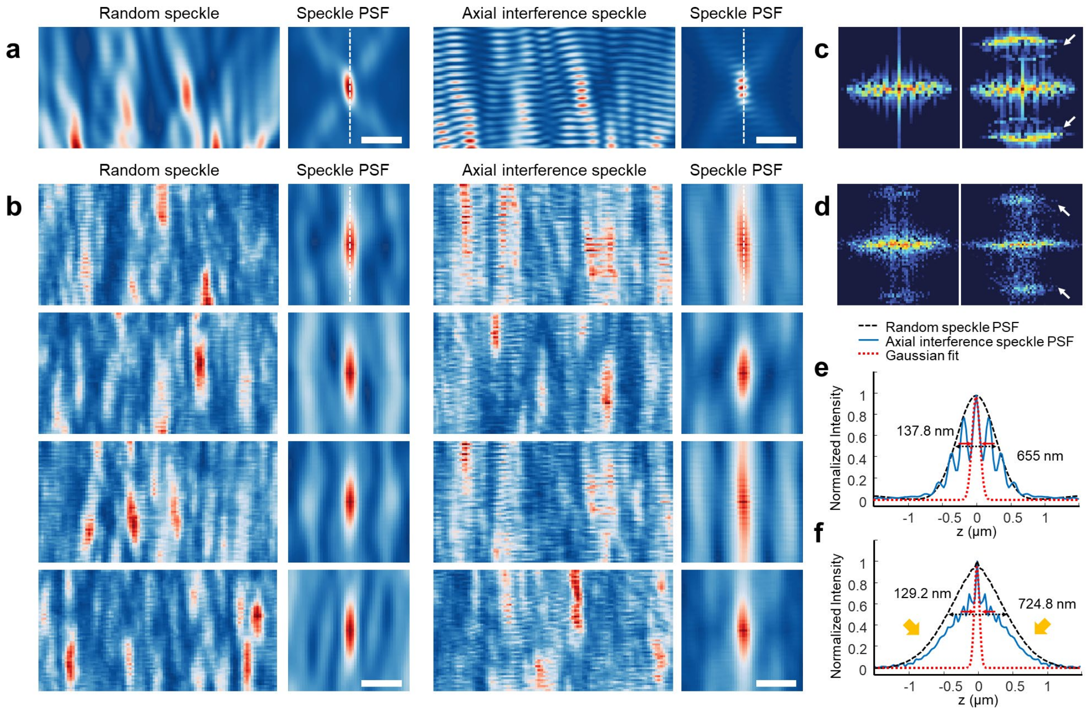

Supplementary Fig. 1 Comparison of FDTD simulations and experimentally captured random speckle and AXIS illumination (a) FDTD simulations of random speckle illumination (left) and AXIS illumination (right). The insets display the effective speckle PSF for each case, defined as the autocorrelation of the product of the FDTD-simulated speckle illumination and the system PSF. Identical to Fig. 1b in the main text. (b) Experimentally captured speckle images (x,z) obtained from random speckle illumination (left) and AXIS illumination (right). These images of the incident light were acquired by replacing the dichroic mirror, which is used for fluorescence signal detection, with a beam splitter. The right insets in each case represent the effective speckle PSF. To acquire these speckle patterns, the diffuser rotation was temporarily halted during image acquisition, thereby reproducing the static condition assumed in the FDTD simulations. (c, d) Fourier domain representations of the speckle patterns shown in (a) and (b). The left and right panels correspond to random speckle and AXIS illumination, respectively. White arrows indicate high-spatial-frequency features that are distinctive of AXIS illumination, highlighting the presence of sufficient axial frequency content. (e, f) Line profiles corresponding to the dashed lines in (a) and (b). The experimentally captured AXIS illumination exhibits z-direction sidelobes in the line profile shown in (f) (yellow arrows), which arise from the influence of the detection PSF incorporated in the effective speckle PSF. Nevertheless, the AXIS illumination still shows reduced axial speckle elongation due to strong central interference in the speckle illumination PSF. Furthermore, although the modulation depth is reduced by the elongated detection PSF, the axial frequency components preserved in (c, d) remain sufficient for effective image reconstruction. As noted in the main text, the volume of the detection PSF in this study was not excessively minimized, as reducing the mirror height too much could compromise the functionality of our system. This includes the wider imaging volume enabled by AXIS illumination, minimal sample damage, and stable mirror alignment. Scale bars: (a, b) 1 μm.

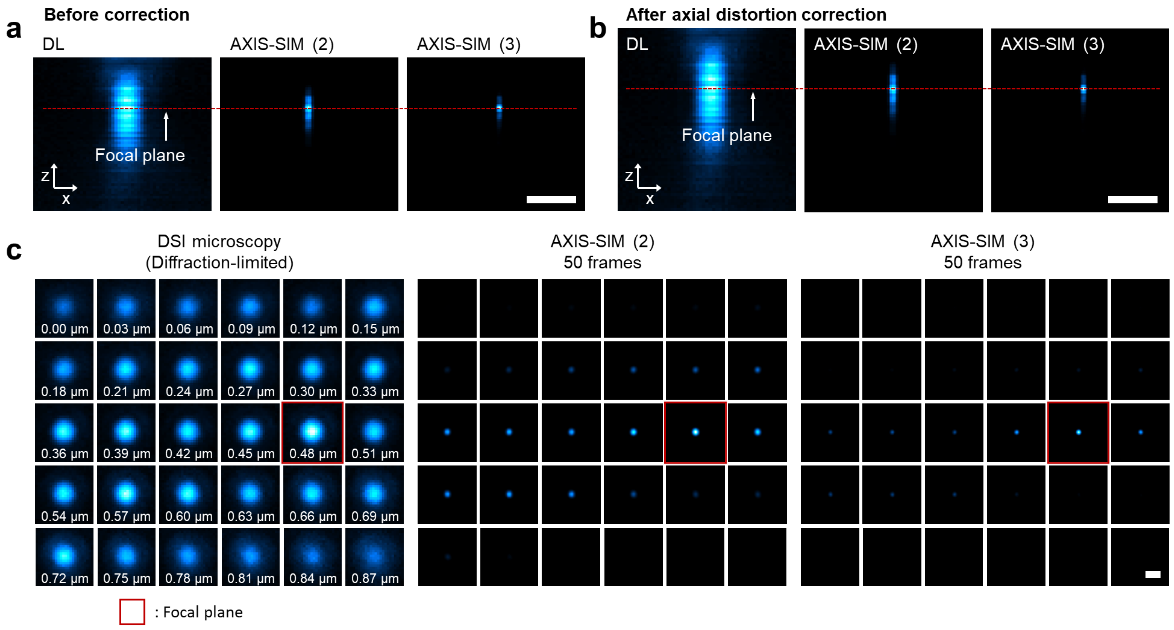

Supplementary Fig. 2 Full data representation of 100 nm bead experiments (refer to main text Fig. 2c). (a, b) Axial cross-sectional views of a single 100 nm bead before (a) and after (b) axial distortion correction using Supplementary Equation (9). (c) Axial montage of the same 100 nm bead, imaged at intervals of 30.1 nm, demonstrating progressive improvements in axial resolution as the reconstruction order increases. From left to right: diffraction-limited DSI image (DL), AXIS-SIM 2nd order (AXIS-SIM (2)) using 50 frames, and AXIS-SIM 3rd order (AXIS-SIM (3)) also using 50 frames. The 30.1 nm axial interval is the result of correcting the initial motorized nanostage interval setting of 25 nm. Scale bars: (a, b) 500 nm; (c) 200 nm.

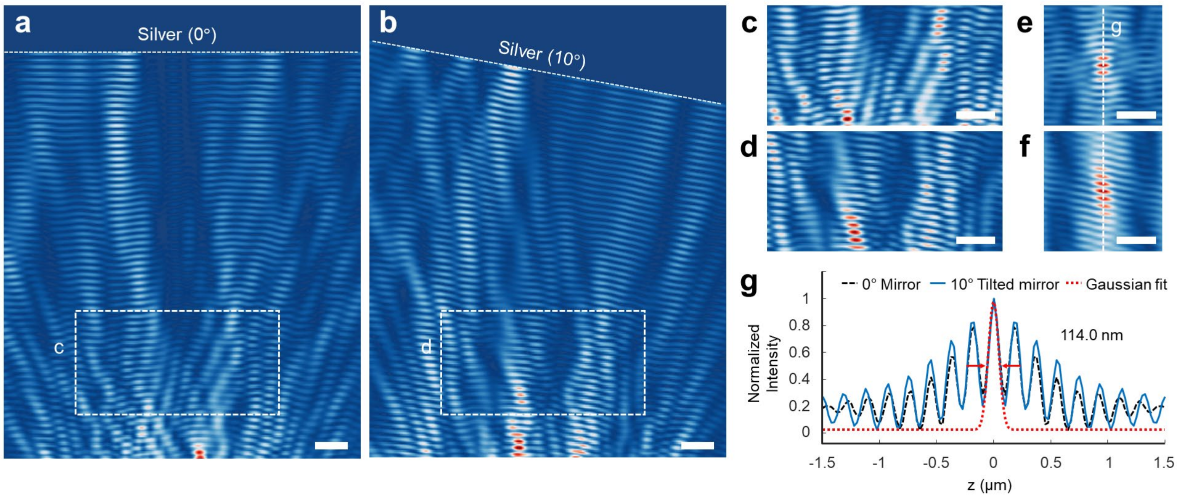

Supplementary Fig. 3 Robustness of AXIS-SIM to mirror angle deviations. (a, b) FDTD simulations comparing a perfectly aligned mirror system (0°) (a) and a mirror tilted by 10° (b). (c, d) Enlarged views of the white dashed boxes in (a) and (b), respectively. (e, f) Speckle illumination PSF obtained by autocorrelating the images from (c) and (d). (g) Line profiles corresponding to white dashed line in (e) and (f), showing that both the 0° and 10° cases maintain a strongly confined z-axis PSF, with no significant differences. Scale bars: (a-f) 1 μm.

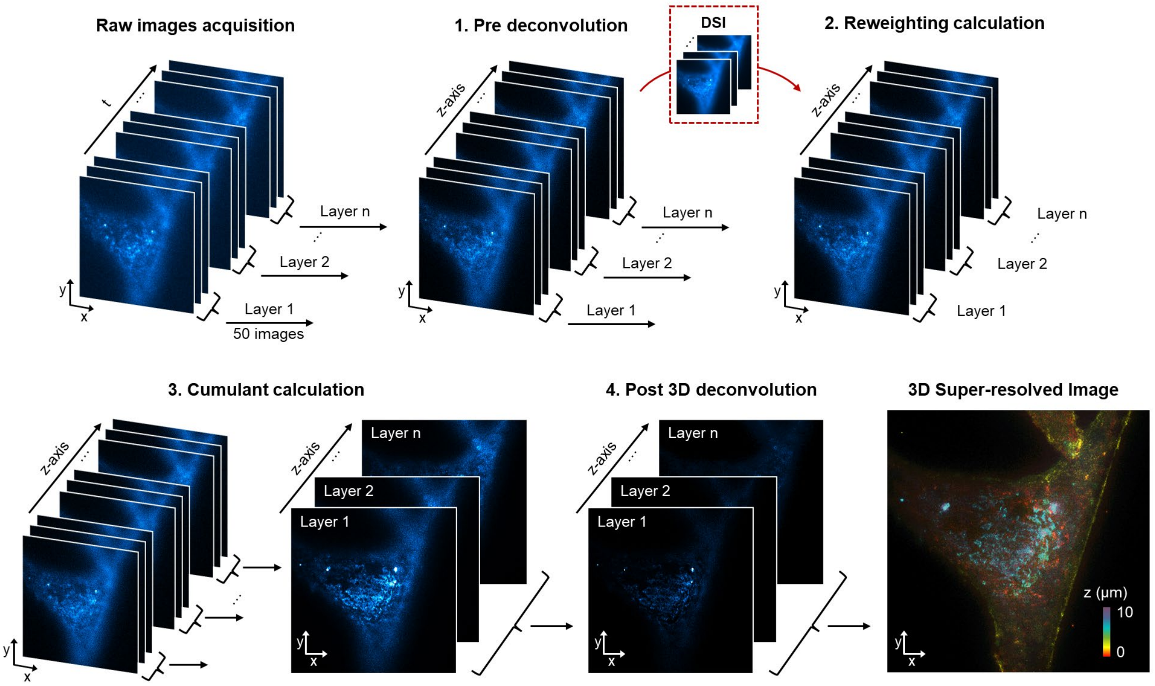

Supplementary Fig. 4 Reconstruction workflow of the AXIS-SIM.

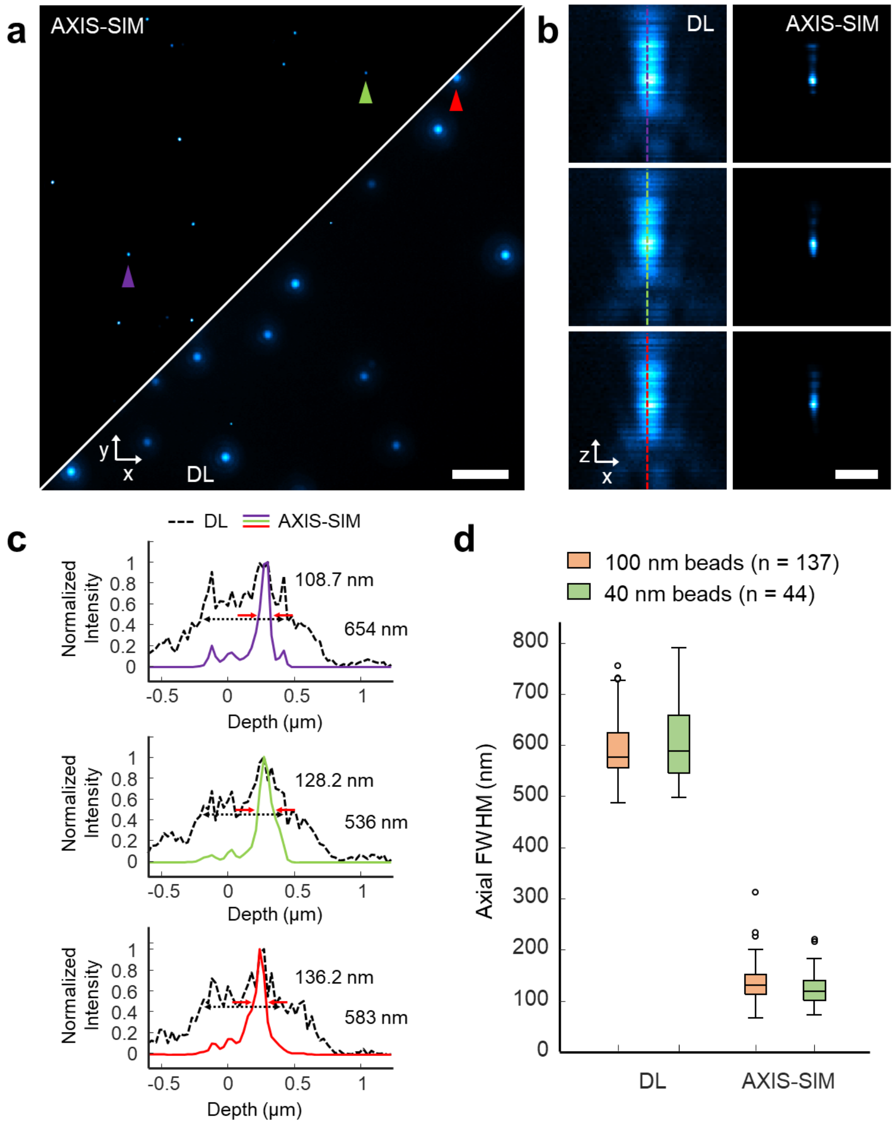

Supplementary Fig. 5 Resolution analysis using 40 nm fluorescent beads. (a) Maximum intensity projection of a DL image and a third-order AXIS-SIM reconstructions of 40 nm fluorescent beads (b) Axial cross-sectional views of three representative beads, corresponding to the colored arrowheads in (a). (c) Line profiles extracted along the dashed lines in (b). (d) Quantification of axial FWHM measurements for 100 nm and 40 nm beads under DL and AXIS-SIM conditions. For 40 nm beads, the average axial FWHM was 125.4 ± 33.5 nm for AXIS-SIM and 609.8 ± 73.0 nm for DL (n = 44). Scale bars: (a) 2 μm; (b) 500 nm.

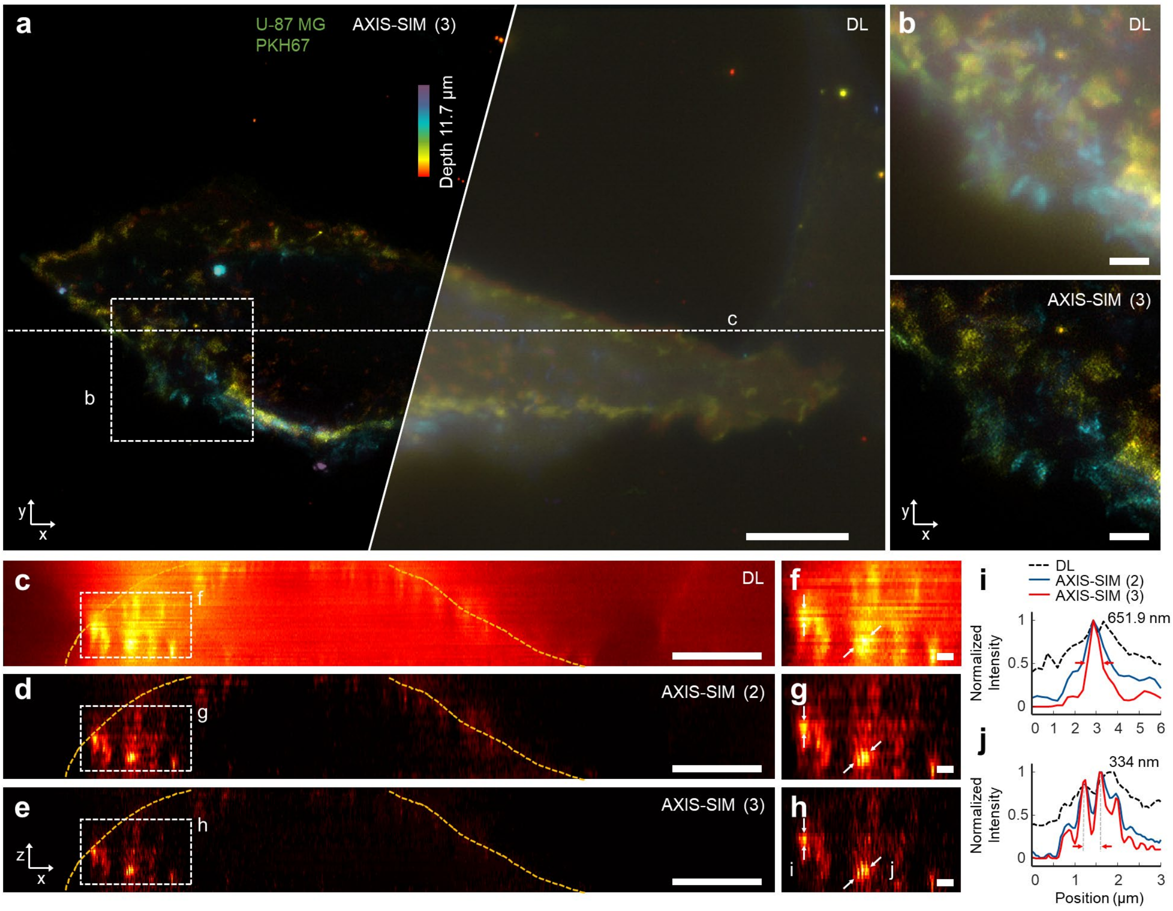

Supplementary Fig. 6 3D resolution enhancement in U-87 MG cell membrane using AXIS-SIM. (a) Comparison of the DL image and super-resolved AXIS-SIM (3) image of the U-87 MG cell membrane. The depth is shown in a color-coded format. Imaging was performed over a total imaging volume of 86.0 × 53.2 × 11.7 μm³ by stitching two ROIs together (Supplementary Fig. 7). (b) Enlarged view of the area inside the white dashed box in (a). (c–e) Axial cross-sectional views along the white dashed line in (a) showing DL, AXIS-SIM (2), and AXIS-SIM (3) images, respectively. The yellow dashed line indicates the cell membrane boundary. (f–h) Magnified versions of the area within the white dashed box in (c–e). (i, j) Line profile for the arrow-marked sections in (f–h), demonstrating enhanced resolving power compared with DL imaging. Scale bars: (a, c–e) 10 μm; (b, f–h) 2 μm.

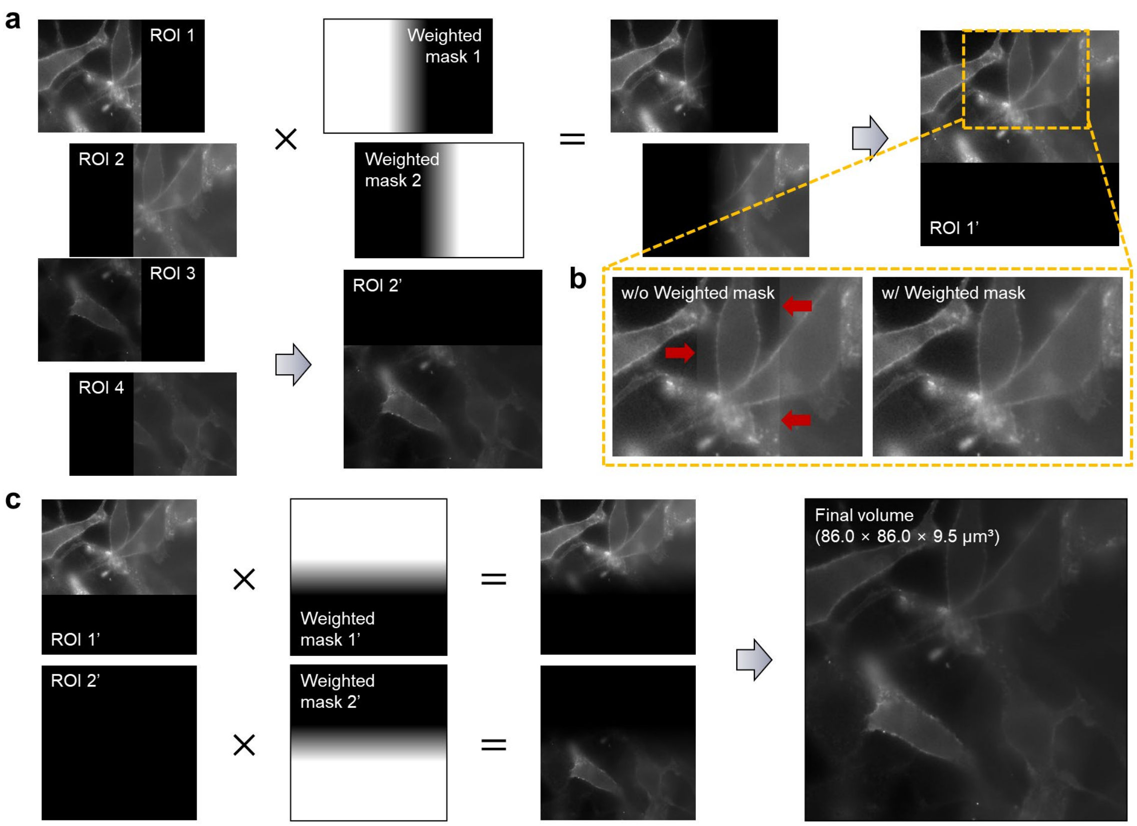

Supplementary Fig. 7 Framework for stitching multiple ROIs to form a larger FOV (a) The process begins with two adjacent regions of interest (ROIs) that overlap, sharing common sample features. Weighted masks are generated for each layer, ensuring that the mask values in the overlapping regions smoothly sum to 1. By multiplying the original ROI with the corresponding weighted mask, a weighted ROI is created. When combined, these weighted ROIs form a larger ROI (ROI'), expanding the field of view (FOV). (b) If stitching is performed without the weighted masks, linear artifacts (indicated by red arrows) appear at the edges where the ROIs meet. However, using weighted masks eliminates these edge artifacts, resulting in a smooth transition between ROIs. (c) The newly created ROI 1' can be stitched with an adjacent ROI 2' using the same weighted mask method, further expanding the FOV. In Supplementary Fig. 6, two ROIs are stitched together to cover an area of 86.0 x 53.2 x 11.7 μm^3. This process can be repeated to achieve an even larger 3D FOV.

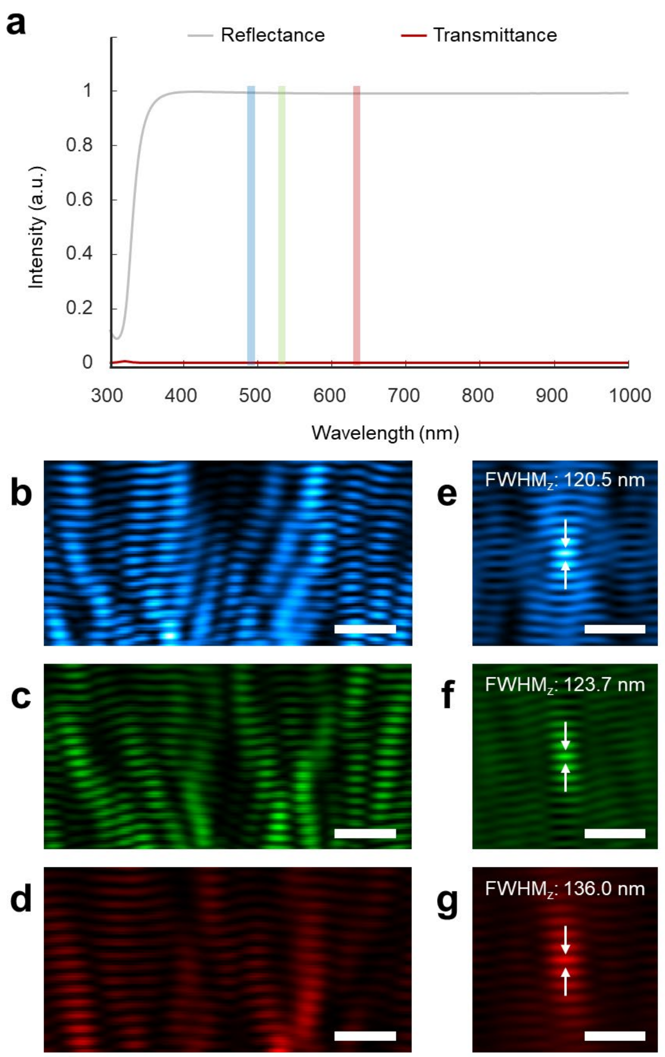

Supplementary Fig. 8 Back-reflecting mirror for multi-wavelength AXIS illumination. (a) FDTD simulation results showing the reflectance and transmittance of the silver mirror at different wavelengths. The reflectance is close to 1 and uniformly distributed throughout the visible to near-infrared regions, enabling AXIS-SIM to be easily applied to multicolor imaging. The refractive index values and the thickness of each deposited layer are consistent with those described in Supplementary Note 1. (b–d) FDTD-simulated patterns of AXIS illumination at wavelengths of 488, 532, and 633 nm, revealing fine axial structures under multi-wavelength AXIS illumination. (e–g) Each image shows the autocorrelation function of the speckle illumination, representing the speckle illumination PSF corresponding to the simulations in (b, c, d), respectively. Scale bars: (b–g) 1 μm.

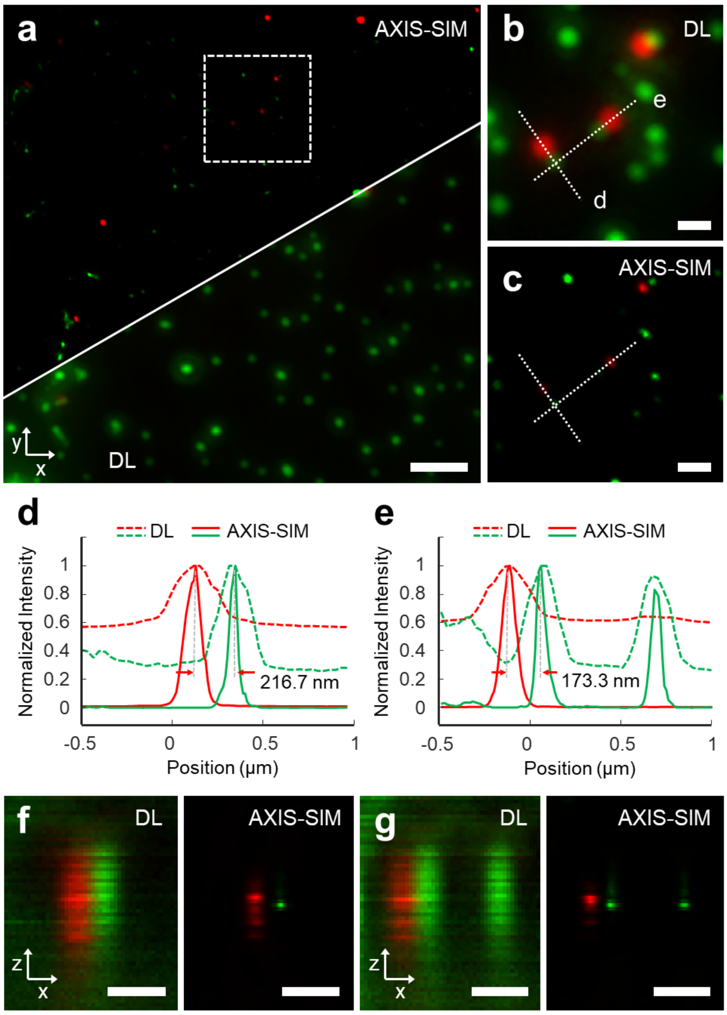

Supplementary Fig. 9 Demonstration of multicolor AXIS-SIM imaging. (a) Maximum intensity projection of a DL and third-order AXIS-SIM images alongside a reconstructed image of a mixture of 200 nm dark-red (660/680 nm) and 100 nm yellow-green (505/515 nm) fluorescent polystyrene beads, illustrating the enhanced resolution capability of AXIS-SIM. A 488 nm laser was used to excite the yellow-green beads, followed by a 633 nm laser for the dark-red beads. (b, c) Enlarged versions of the area within the white dashed box in (a) for DL (b) and AXIS-SIM (3) (c). (d, e) Line profiles along the white dashed lines in (b) and (c), showing resolution enhancement for both dark red and yellow-green beads under each imaging condition. (f, g) Axial views along the white dashed lines in (b) and (c) provide detailed cross-sectional representations of the beads. Scale bars: (a) 2 μm; (b, c, f, g) 500 nm.

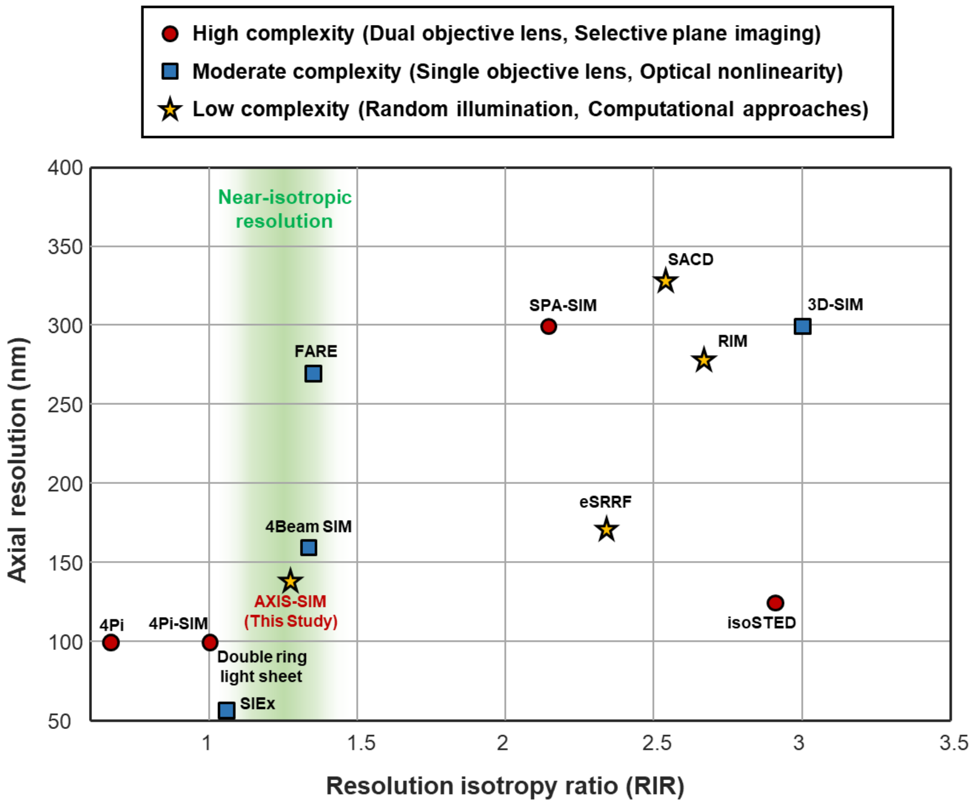

Supplementary Fig. 10 Comparative analysis of various super-resolution imaging techniques by plotting their axial resolution against the resolution isotropy ratio (RIR), based on the data in Supplementary Table 3. Imaging techniques are categorized by instrumental complexity: high (●, dual objective lens, selective plane imaging), moderate (■, single objective lens, optical nonlinearity), and low (★, random Illumination, computational approaches). AXIS-SIM achieves near-isotropic resolution (RIR of 1.29) with minimal instrumental complexity, as highlighted in the green-shaded region.

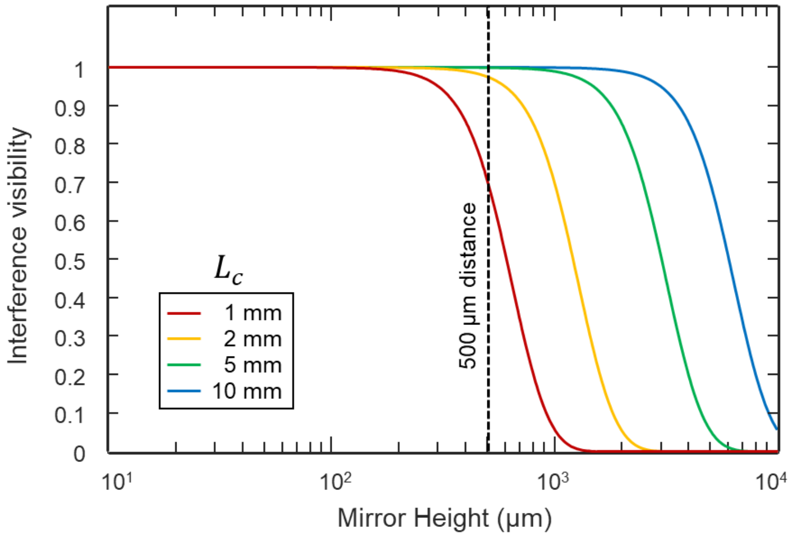

Supplementary Fig. 11 Calculated interference visibility as a function of mirror height for different coherence lengths.

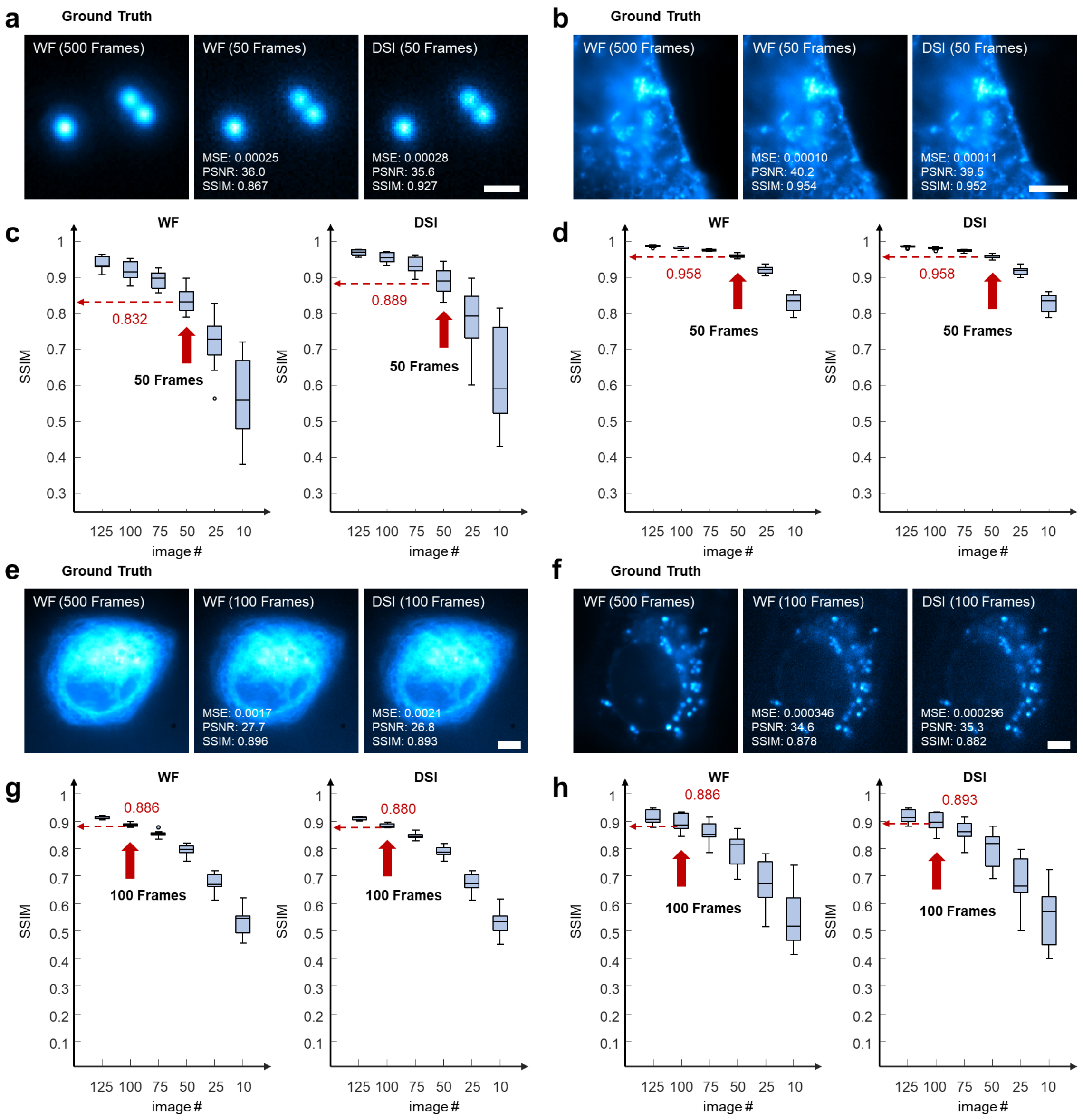

Supplementary Fig. 12 Comparisons of image quality based on the number of frames (a) The left image shows the sum of 500 raw images of 100 nm beads using AXIS illumination, which serves as the ground truth (GT). The middle image displays the sum of 50 raw frames using AXIS illumination, while the right image was generated by calculating the RMS of 50 frames using DSI. (b) In a similar comparison for fixed U-87 MG cell membrane images, the left image shows the sum of 500 raw frames as GT, the middle shows the sum of 50 raw frames, and the right is created using DSI from 50 frames. (c) Image quality for 100 nm beads was performed by randomly selecting 125, 100, 75, 50, 25, and 10 frames from the full set of 500 and comparing their structural similarity (SSIM) to the GT. As the number of frames decreases, structural similarity drops significantly. However, the DSI-reconstructed image from only 50 frames still showed high similarity to the GT (about 0.9). This indicates that DSI, even with fewer frames, provides better optical sectioning compared to wide-field imaging (WF), consistent with prior research[1]. (d) A similar analysis was done for cell images, where randomly selected sets of 125, 100, 75, 50, 25, and 10 frames were compared to the GT using SSIM. Even with the reduction in frames, the DSI image from 50 frames remained highly similar to the GT (around 0.95). Based on these results, 50 frames

were selected for the main study, offering a balance between efficiency and image quality. (e–h) In live U2OS cell imaging of microtubules (e, g) and lysosomes (f, h), similar trends to those observed in fixed samples (a–d) were noted. However, for live cell experiments, 100 frames per layer were used to ensure high SSIM values. For stationary samples, such as beads and fixed cells, 50 frames per z-layer were sufficient to achieve a robust signal-to-noise ratio (SNR) and high-fidelity reconstructions. In contrast, live cell imaging required an additional 50 frames per layer to compensate for the dynamic nature of organelles to maintain resolution and SNR. Preliminary tests showed that 100 frames per layer achieved an optimal balance between capturing cellular dynamics at high resolution while minimizing phototoxicity. Scale bars: (a) 500 nm; (b, e, f) 5 μm.

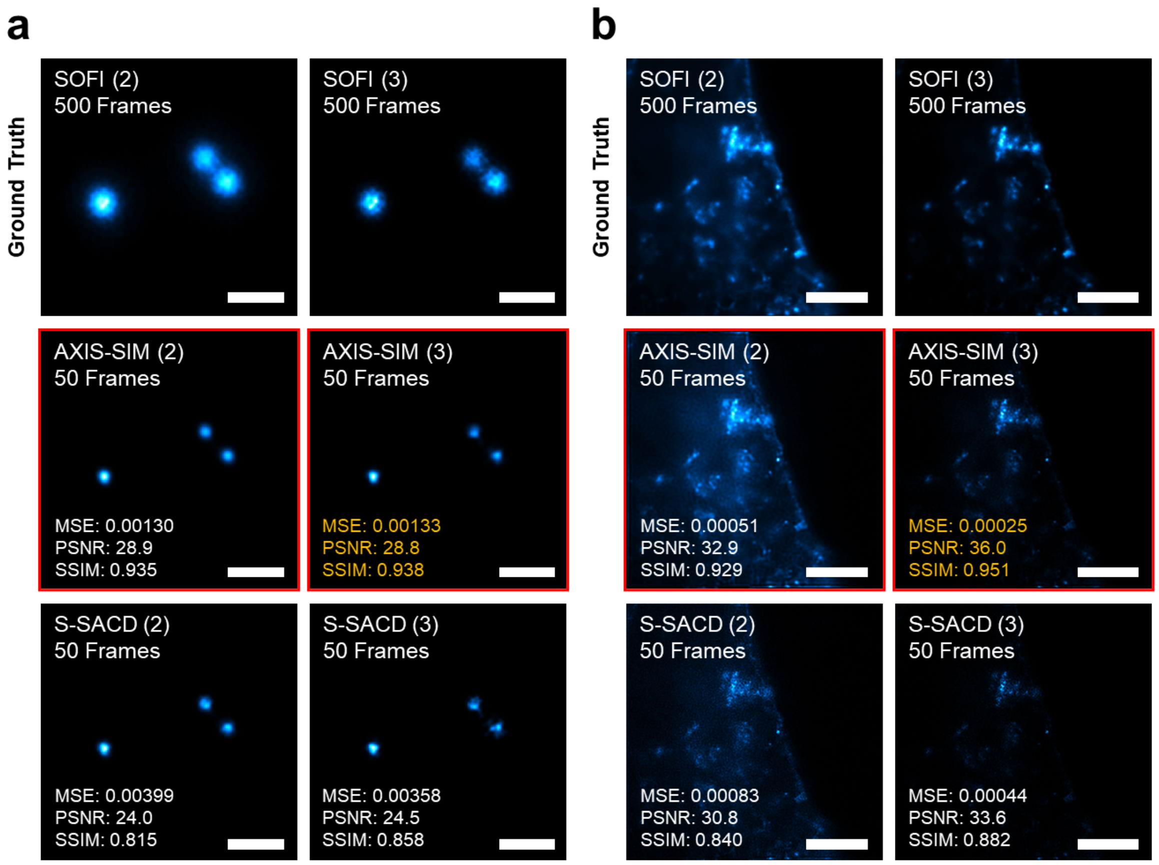

Supplementary Fig. 13 Comparisons of reconstructed image quality based on the order of cumulant calculations and the number of frames. (a) The figure displays 500-frame SOFI (2nd and 3rd order) images of beads, which serve as the GT. In comparison, 50-frame AXIS-SIM (2nd and 3rd order) images, as well as 50-frame SACD (2nd and 3rd order) images, are shown. Since SACD was calculated using speckle images instead of wide-field illumination, we refer to this as S-SACD (speckle-SACD) to avoid confusion. Below each image, MSE, PSNR, and SSIM values are provided, comparing them to the 500-frame SOFI image (GT). Notably, AXIS-SIM (3rd order) produced bead images with comparable or even superior quality than the 500-frame SOFI images. (b) A similar analysis was conducted for the cell membrane images. These results solidify our decision to use 50 frames in combination with 3rd order AXIS-SIM reconstructions for the main study, as this approach offers an optimal balance between efficiency and image quality. Scale bars: (a) 500 nm; (b) 5 µm.

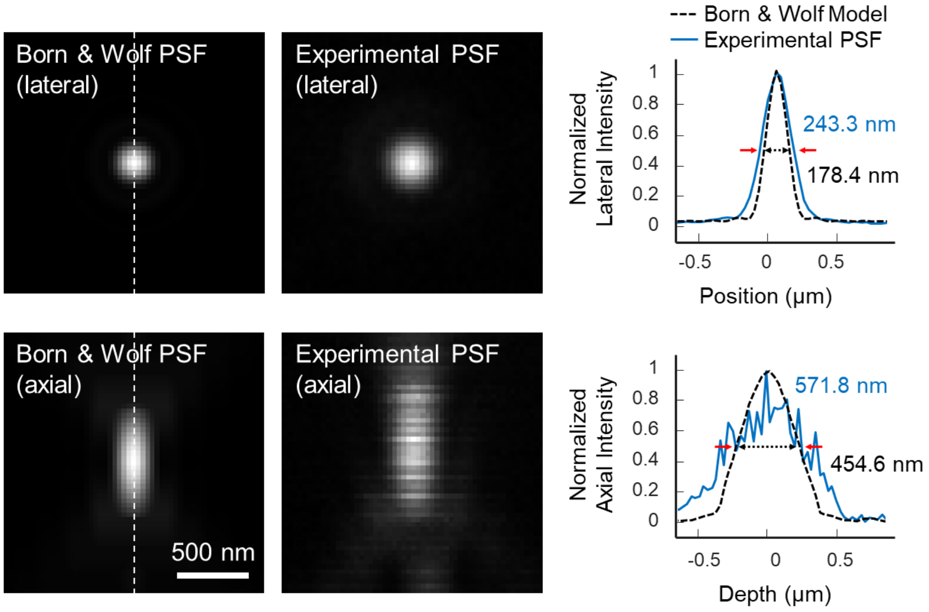

Supplementary Fig. 14 Comparison of the theoretical PSF generated using the Born–Wolf model and the experimentally measured PSF from 40 nm fluorescent beads. Normalized lateral and axial line profiles showing FWHM values of 243.3 nm vs. 178.4 nm (lateral) and 571.8 nm vs. 454.6 nm (axial) for experimental and theoretical PSFs, respectively. Despite the slightly broader profiles and modest asymmetries in the experimental data, the core features of the PSF—such as lateral width and axial elongation—are well preserved, supporting the use of theoretical PSF for deconvolution.

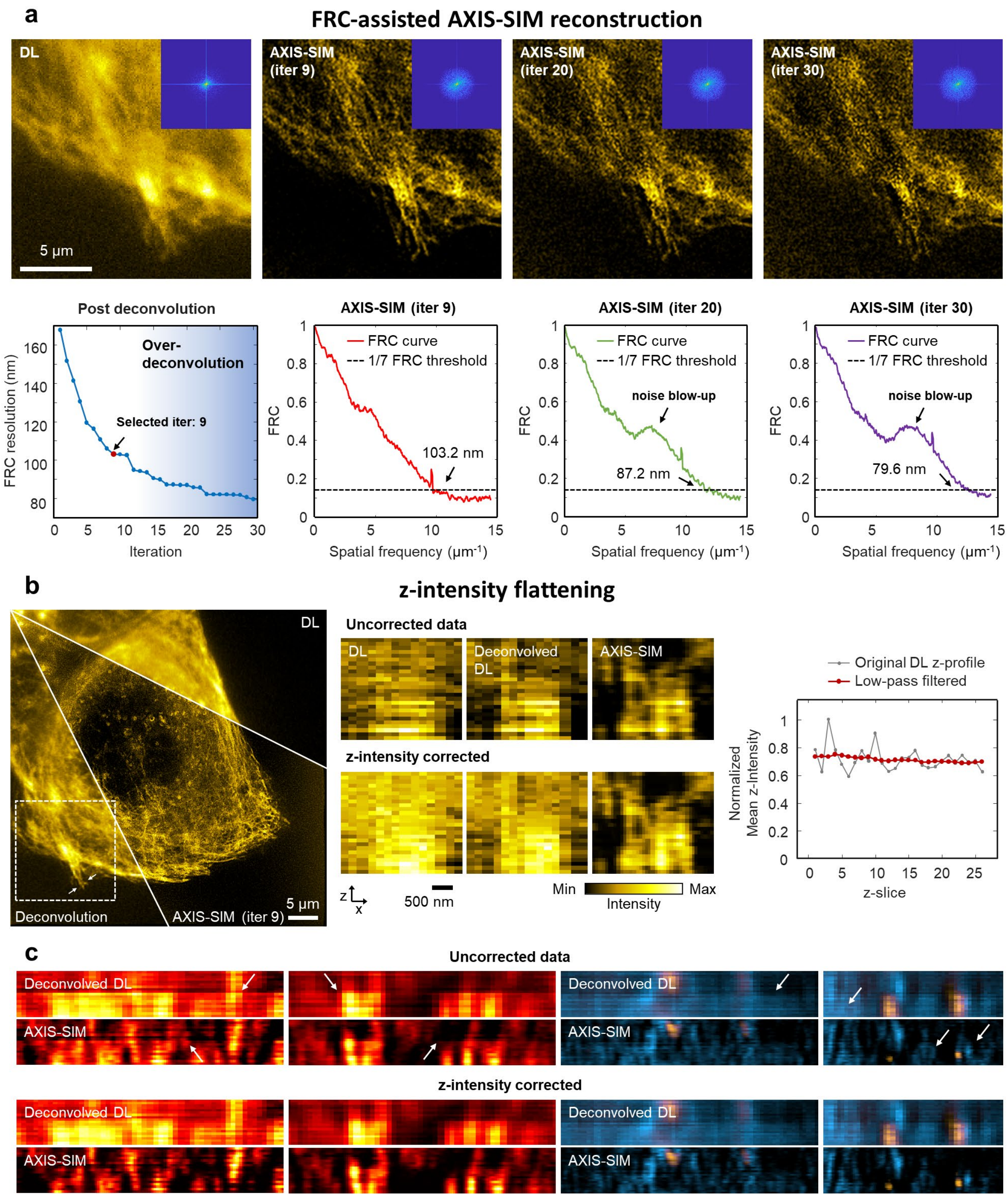

Supplementary Fig. 15 Enhancing AXIS-SIM with FRC-assisted deconvolution and z-intensity flattening. (a) FRC-assisted AXIS-SIM reconstruction of microtubules. Top row: DL image and AXIS-SIM reconstructions obtained with different RL iteration numbers (9, 20, and 30). Insets show the corresponding 2D Fourier spectra. Bottom left: FRC-estimated resolution as a function of RL iteration; the red dot marks the objectively selected iteration (iteration = 9) before over-deconvolution sets in. Bottom middle/right: FRC curves (solid lines) for iteration = 9, 20, and 30, together with the 1/7 FRC threshold. The FRC-derived resolutions were 103.2 nm (iteration = 9), 87.2 nm (iteration = 20), and 79.6 nm (iteration = 30), but the latter two exhibit evident high-frequency noise blow-up and were therefore rejected by our stopping criterion. (b) z-Intensity flattening.

Left: Maximum intensity projection of DL image, deconvolved DL, and AXIS-SIM (iteration = 9) after z-intensity correction. Center: Enlarged x–z cross-sections (locations marked by white arrows) showing uncorrected (top) versus z-flattened (bottom) data. Right: Mean z-intensity profiles of the original DL volume (gray) and the low-pass-filtered curve (red). A correction factor computed as the ratio at each depth between the original and filtered profiles was applied to every z-slice to produce the final flattened images. (c) Representative examples of z-intensity flattening on main text figures. Left panels: data from main text Fig. 3d, e before and after correction. Right panels: data from main text Fig. 4e, f before and after correction. White arrows indicate stripe artifacts that are substantially reduced following z-flattening.

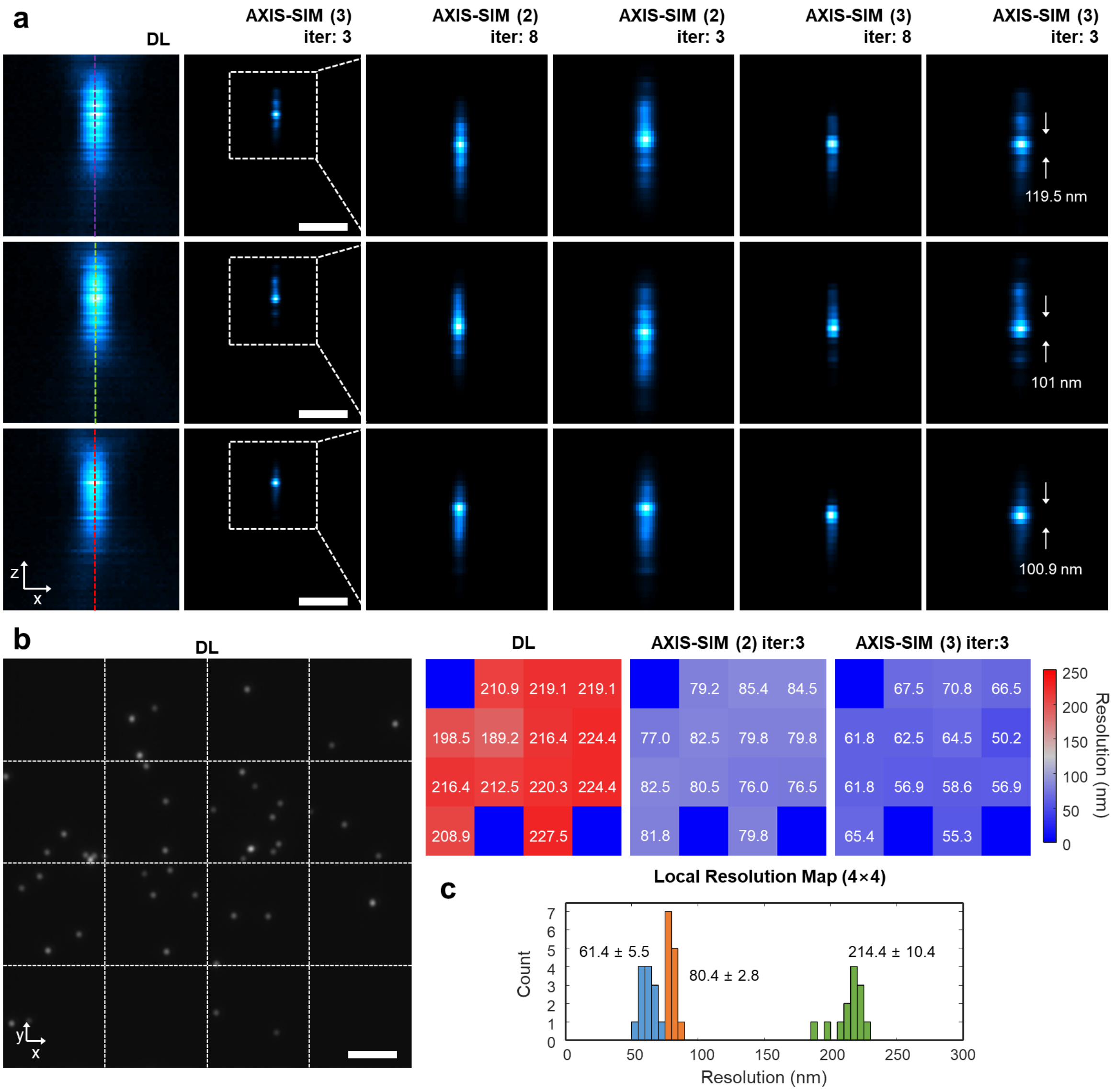

Supplementary Fig. 16 Artifact suppression and spatial uniformity in AXIS-SIM reconstruction. (a) Axial cross–sections of individual 100-nm beads acquired in DL and in second-order and third-order AXIS-SIM (denoted AXIS-SIM (2) and AXIS-SIM (3), respectively). For each order, results are shown after 3 and 8 RL-deconvolution iterations. Although the bead FWHM does not directly represent system resolution, applying FRC-assisted iteration stopping (iter = 3) successfully alleviated lateral over-minimization while preserving axial confinement (~100–120 nm FWHM), resulting in more spherical bead morphology in 3D. The dataset AXIS-SIM (3) iter 8, corresponds to that shown in Fig. 2c–e of the main text. (b) Maximum intensity projection of the DL bead volume, subdivided into a 4 × 4 grid. Local lateral resolution in each tile was estimated using decorrelation analysis. The resulting resolution maps for DL, AXIS-SIM (2), and AXIS-SIM (3) at iter = 3 show consistent improvements with increasing order and minimal spatial variation across the field of view. (c) Histogram of local resolution values from the 13 central tiles in (b). Mean ± s.d. are indicated for each dataset. The narrow and consistent distributions suggest that the reconstruction process preserves spatial uniformity and approximate shift invariance. Scale bars: (a) 500 nm; (b) 2 µm.

Supplementary Tables

Supplementary Table 1 | The full dataset of lateral and axial FWHM of the effective speckle PSF in main text Fig. 1d. The table provides statistical analysis of n = 101 speckle images, std: standard deviation.

	**Random speckle**		**Axial interference speckle (AXIS)**	
**FWHM (nm)**	**Mean**	**std**	**Mean**	**std**
Lateral	322.39	28.89	703.48	21.00
Axial	376.99	29.02	**156.63**	**17.25**

Supplementary Table 2 | The full dataset of axial FWHM measurements of 100 nm beads in main text Fig. 2k. The table provides statistical analysis of n = 137 bead images, analyzed under three systems: diffraction-limited DSI, AXIS-SIM (2nd order), and AXIS-SIM (3rd order), std: standard deviation.

		**Random speckle**		**Axial interference speckle (AXIS)**	
**Imaging system**	**FWHM (nm)**	**Mean**	**std**	**Mean**	**std**
DL	Axial	568.50	77.47	591.91	54.80
AXIS-SIM (2)	Axial	214.28	51.38	216.47	42.58
AXIS-SIM (3)	Axial	140.46	48.39	**133.45**	**34.54**

Supplementary Table 3 | Overview and comparison of previous axial super-resolution techniques with this study.

Year	Microscopy Configuration	Illumination Type	Key Instruments	Lateral Resolution (nm)	Axial Resolution (nm)	Resolution Isotropy Ratio (RIR)	Required Frames (only wide-field acquisition)	Ref.
**1992**	4Pi	Point scanning	Dual objective	~100-150	~100	0.67	-	[11]
**1999**	i5M	Wide-field	Dual objective	Diffraction-limit	~100	-	-	[12]
**2008**	isoSTED	Point scanning	Dual objective	43	125	2.91	-	[13]
**2008**	3D SIM	Wide-field	Structured illumination	~100	~300	3.00	15 frames per layer	[22]
**2010**	ISO	Point scanning	**Mirror**	Diffraction-limit	~200	-	-	[17]
**2016**	MEANS	Point scanning	**Mirror**	Diffraction-limit	~100	-	-	[18]
**2021**	SELFI + mirror	Wide-field	**Mirror** with PSF pattern comparison	Diffraction-limit	~3	-	-	[19]
**2021**	RIM	Wide-field	Speckle illumination, **Computational reconstruction**	105	280	2.67	800 frames per layer	[25]
**2022**	Double ring light sheet	Selective plane illumination	Double ring Bessel beam, Deep learning	~100	~100	1.00	-	[14]
**2023**	eSRRF	Wide-field	**Computational reconstruction**	~74	~173	2.34	1000 frames per layer	[9]
**2023**	4Beam SIM	Wide-field	**Mirror** with structured illumination	~120	~160	1.33	15 frames per layer	[20]
**2023**	SACD	Wide-field	**Computational reconstruction**	~130	~330	2.54	20 frames per layer	[34]
**2024**	SPA-SIM (two-photon)	Selective plane illumination	Selective plane activation with photoswitchable fluorescent proteins	~140	~300	2.14	9–15 frames per layer	[15]
**2024**	SIEx	Point scanning	**Mirror** with optical nonlinearity	54	57	1.06	-	[21]
**2024**	FARE	Wide-field	Structured illumination	~200	~270	1.35	3 frames per layer	[23]
**2025**	4Pi-SIM	Wide-field	Dual objective with structured illumination	~100	~100	1.00	15 frames per layer	[16]
**-**	**AXIS-SIM**	Wide-field	**Mirror with speckle illumination, Computational reconstruction**	~110	~140	1.29	50–100 frames per layer	**This study**

Supplementary Table 4 | PSF calculation parameters used in AXIS-SIM reconstruction (n: refractive index).

Experiment	NA	Wavelength (nm)	n_{sample}	Pixel size xy (nm)	Nanostage z-step (nm)	Corrected z-step (nm)
100 nm Beads (Fig. 2, Supplementary Fig. 9)	1.49	515	Water (n = 1.33)	32.5	25	30.01
200 nm Beads (Supplementary Fig. 9)	1.49	680	Water (n = 1.33)	32.5	25	30.01
U2OS Cell Microtubules (Fig. 3, 4, Supplementary Fig. 15)	1.49	515	PBS (n = 1.338)	104	40	47.60
U2OS Cell Lysosomes (Fig. 4, 5)	1.49	680	PBS (n = 1.338)	104	40	47.60
U-87 MG Cell (Fig. 3)	1.49	515	PBS (n = 1.338)	104	40	47.60
U-87 MG Cell (Supplementary Fig. 6)	1.49	515	PBS (n = 1.338)	104	200	238.01

Supplementary Table 5 | Quantitative evaluation of resolution change before and after z-intensity flattening.

**Microtubule Cases**	**Axial resolution (Uncorrected, nm)**	**Axial resolution (z-intensity flattened, nm)**	**Resolution difference Δ (%)**
Supplementary Fig. 15b	145.58	145.45	0.09
Fig. 3d	147.51	147.51	0.00
Fig. 3e	146.73	147.71	0.66
Fig. 4e	145.97	145.97	0.00
Fig. 4f	144.57	144.25	0.22

Supplementary References

1. Palik, E. D. Handbook of Optical Constants of Solids. (Academic Press, 1985).
2. Johnson, P. B. & Christy, R. W. Optical constants of the noble metals. *Phys. Rev. B* **6**, 4370–4379 (1972).
3. Ventalon, C. & Mertz, J. Quasi-confocal fluorescence sectioning with dynamic speckle illumination. *Opt. Lett.* **30**, 3350–3352 (2005).
4. Diel, E. E., Lichtman, J. W. & Richardson, D. S. Tutorial: avoiding and correcting sample-induced spherical aberration artifacts in 3D fluorescence microscopy. *Nat. Protoc.* **15**, 2773–2784 (2020).
5. Goodman, J. W. Statistical Optics, 2nd ed. (Wiley, 2015).
6. Li, X. *et al.* Three-dimensional structured illumination microscopy with enhanced axial resolution. *Nat. Biotechnol.* **41**, 1307–1319 (2023).
7. Zhao, W. *et al.* Enhanced detection of fluorescence fluctuations for high-throughput super-resolution imaging. *Nat. Photonics* **17**, 806–813 (2023).
8. Liu, Y., Panezai, S., Wang, Y. & Stallinga, S. Noise amplification and ill-convergence of Richardson-Lucy deconvolution. *Nat. Commun.* **16**, 911 (2025).
