# Supplementary material for: Near-isotropic super-resolution microscopy with axial interference speckle illumination
Source: Nat Commun. 2025 Oct 20;16:9274. doi: 10.1038/s41467-025-64366-2 (PMC12537971; doi:10.1038/s41467-025-64366-2)
Supplement: Supplementary file 2 — Description of Additional Supplementary Files [file 41467_2025_64366_MOESM2_ESM.pdf]

Description of Additional Supplementary Files

Supplementary Movie 1

Comparison of diffraction-limited DSI image (DL), 3D RL-deconvolved DL results, and super-resolved AXIS-SIM image of live U2OS cell microtubules. This movie shows the maximum intensity profiles along the z-axis and provides comparisons across different ROIs. See also Fig. 3a–h.

Supplementary Movie 2

3D projections of fixed U-87 MG cells labeled for membranes, comparing the 3D RL-deconvolved DL image and the corresponding AXIS-SIM image. See also Fig. 3i–o for details.

Supplementary Movie 3

3D projections from two-color imaging of live U2OS cells, visualizing both microtubules and lysosomes. This movie compares the 3D RL-deconvolved DL image with the super-resolved AXIS-SIM image. See also Fig. 4a, b.

Supplementary Movie 4

3D projections of live U2OS cell lysosomes, comparing the 3D RL-deconvolved DL image and the corresponding AXIS-SIM image. See also Fig. 5a.

Supplementary Movie 5

Time-lapse imaging of live U2OS cells stained to highlight lysosome distribution within a single focal plane. The movie compares the RL-deconvolved DL images with the AXIS-SIM images, acquired at 2.0-s intervals over 15 time points. See also Fig. 5h–l for details.

Supplementary Movie 6

Additional time-lapse imaging of live U2OS cells stained to highlight lysosome distribution within a single focal plane. The movie compares the RL-deconvolved DL images with the AXIS-SIM images, acquired at 2.0-s intervals over 30 time points.
